# Analysis on the characteristics and relationships of lower limbs strength and power of sailors in different positions and levels

**DOI:** 10.1371/journal.pone.0289273

**Published:** 2023-08-18

**Authors:** Kaiyang Sun, Dandan Pan

**Affiliations:** 1 Shanghai Research Institute of Sports Science, Shanghai, China; 2 Shanghai Elite Sport Training Administrative Center, Shanghai, China; King Khalid University, INDIA

## Abstract

Hikers and trapeze sailors are two main Olympic groups based on their specific role during sail racing. This study was to analyze the characteristics and relationships of lower limbs strength and power of hikers and trapeze sailors with different levels. Forty-five hikers and trapeze sailors were divided into high-level and low-level groups, respectively. The isokinetic dynamometer was used to measure sailors’ lower limbs strength. Concentric and eccentric peak torque (PT) were measured for hamstrings and quadriceps at 60°/s, and isometric knee extensions at 45° knee flexion (0° = full extension). The counter movement jump (CMJ) was performed by Kistler to reflect sailors’ lower limbs power. The results showed the hikers had higher quadriceps isometric PT, and male hikers had higher concentric and eccentric PT compared to trapeze sailors (*p*<0.05). For female sailors, the quadriceps’ concentric, eccentric and isometric PT of high-level group were higher than low-level group’ (*p*<0.05). The sailors’ conventional and functional H/Q ratio ranged from 0.44 to 0.56 and 0.52 to 0.65, respectively. High-level and low-level groups had no difference in CMJ, which was moderately to strongly correlated with the isokinetic strength (r ranging 0.50–0.81, *p*<0.01). These findings suggest that long-term training can induce specificity in lower limbs strength of sailors. Hikers have better quadriceps isometric strength compared to trapeze sailors. The quadriceps concentric and eccentric PT can be used as parameters to distinguish between high-level and low-level sailors, but CMJ cannot.

## Introduction

Sailing is a complex water sport in which the sailors use their body weight, position changes, a series of maneuvers and equipment adjustments to maintain the balance of the hull and optimal speed according to the environmental conditions. Due to the difference of boat type and sailor position, the physical demands, fitness characteristics and technical maneuvers of sailors are different [1]. Two main groups of Olympic class sailors were identified [2, 3]: (1) hikers, including ILCA6, ILCA7, 470 helmsmen, who are placing the foot in hiking strap that is located in the center of the boat, sitting on the deck and leaning over the side of boat; (2) trapeze sailors, including 49er, 49erFX, Nacra 17, 470 crew, who place their foot on the edge of the boat wings supported by a wire that is extended from the rigging. The purposes of the sailors in both positions are to counter the tilting moment generated by the wind on the sails and correct the position of the boat to improve its speed and performance. The movement patterns and postures maintenance of hiking and trapezing require strong strength and power support [4, 5], so it is necessary to evaluate the performance profiles of lower limbs strength and power in sailors.

The CMJ test and isokinetic dynamometer of knee joint are widely used on athletes to evaluate the lower limbs power and muscle strength [6]. CMJ is the action of the human body under the control of the central nervous system, relying on the coordination of all links of the body to exert the maximum explosive force of the lower limbs muscle group, which can reflect the multiarticular ability to generate muscle–tendon unit power. However, CMJ test could not verify the bilateral strength difference of the same muscle or different muscles within the limb, since other muscle groups might compensate these differences [7]. Isokinetic dynamometer can measure muscle strength parameters of single joint or joint chains under different motion modes and angular velocities, and provide a more detailed assessment of the torque generating capacity of the muscles involved in specific joint movements. The combination of the two can better reflect the muscle strength and power characteristics of athletes [6, 8, 9].

The aims of this study were to analyze the characteristics and relationships of lower limbs strength and power between hikers and trapeze sailors with different levels. It was hypothesized that measures of PT of knee flexors and extensors (hamstrings and quadriceps), CMJ performance (height, peak force and peak power) would differentiate training levels of hikers and trapeze sailors.

## Material and methods

### Participants

This study included 45 well-trained sailors (22 male: age = 21.0±3.7 years, height = 182.2±6.9 cm and weight = 73.9±8.6 kg; 23 female: age = 20.6±4.1 years; height = 170.5±6.1 cm and weight = 63.0±7.4 kg), who were divided into two groups: hikers (including 9 ILCA6, 8 ILCA7, 9 470 helmsmen) and trapeze sailors (including 7 49er, 5 49erFX, 7 470 crew). All the sailors have more than 3 years sailing and resistance training experience, and training mean time is higher than 25 hours per week. Exclusion criteria were previous diagnosis of inflammatory or metabolic diseases, only data collected on sailors free of injury and illness are reported. The demographic information and training age of subjects were shown in Table 1. According to the athletes’ grades level, the two groups of sailors were divided into the high-level group and low-level group. The high-level group consisted of international or national level athletes, and the low-level group consisted of athletes of other grades level (more than 3 years of sailing training experience). All the participants were informed about the risks and benefits, informed consent was obtained before commencement of the study. The study protocols were approved by the Capital University of Physical Education and Sports Ethics Committee and according to the ethical principles of the World Medical Association Declaration of Helsinki.

**Table 1:** Demographic information and training age for sailors.

Group	Level	Gender	Height (cm)	Weight (kg)	Age (year)	Training Age (year)
Hikers	High-level	M (N = 6)	178.2±7.6	67.9±8.3	22.0±3.8	11.7±6.0
		F (N = 5)	166.1±4.9	59.6±5.4	23.8±3.5	14.2±4.0
	Low-level	M (N = 5)	180.8±1.7	73.8±4.7	17.5±0.6	3.6±0.5
		F (N = 10)	169.5±6.7	59.4±6.1	17.6±2.1	4.8±1.6
Trapeze sailors	High-level	M (N = 5)	178.8±5.1	75.0±6.3	24.6±4.3	12.2±3.3
		F (N = 4)	174.0±6.4	65.3±3.4	25.2±4.0	14.5±2.4
	Low-level	M (N = 6)	189.7±5.4	79.2±11.2	19.4±1.2	5.0±2.1
		F (N = 4)	175.0±1.8	74.3±4.6	17.0±2.2	3.5±1.0

Note: M: male; F: female.

### Procedures

#### Isokinetic dynamometry

The isokinetic dynamometer (Isomed 2000, D&R Ferstl GmbH, Germany) was used to measure knee muscles strength. Before the test, athletes performed 10–15 minutes warm-up, which consisted of pedaling at 60–80 rmp on a Wattbike ergometer and dynamic stretching concentrating on the lower limbs. During the test, the athlete was required to sit on Isomed chair and use safety belts to secure the torso, hips and distal thigh. The knee joint rotation axis coincided with the rotation axis of the dynamometer. The athletes performed five consecutive maximum concentric and eccentric contractions for hamstrings and quadriceps at angular velocities of 60°/s. After isokinetic test, three 5 sec maximal unilateral isometric knee extensions at 45° knee flexion (0° = full extension) were performed by athletes [10]. Each test was interspersed with 2-minute of rest. The following data were analyzed: quadriceps/hamstrings concentric and eccentric PT, quadriceps isometric PT, hamstrings concentric PT/ quadriceps concentric PT (conventional H/Q ratio), hamstrings eccentric PT/ quadriceps concentric PT (functional H/Q ratio). To achieve rational comparison of muscles strength between hikers and trapeze sailors, we expressed strength in relative values (normalized to body mass).

#### CMJ test

The Kistler MARS (5695BQ2, Kistler Instrumente, Switzerland) was used to measure CMJ performance. Before the test, the athlete performed 8–10 minutes dynamic warm-up, which consisted of squat, lunge, the greatest stretch and progressive jogging exercises. The athlete performed from the upright standing position with their hands on the hips, flexed the hips and knees after hearing the start of the tone, immediately followed by extension of these joints and jump as high as possible [11]. Three CMJ were performed at a 2-minute interval for each test. Jump height, peak force and peak power were recorded (normalized to body mass).

### Statistical analysis

All data were presented with mean ± standard deviation and analyzed with the SPSS Statistics V26.0 software. Mann Withney U test was used to compare the isokinetic dynamometry and CMJ test of lower limbs between hikers and trapeze sailors with different levels. Spearman Correlation Analysis was used to verify the relationship among the data concerning quadriceps PT and CMJ performance (the quadriceps PT value is the sum of the left and right PT). A significant level was accepted at the 95% confidence level for all statistical parameters (*p*<0.05).

## Results

The isokinetic strength of quadriceps and hamstrings and CMJ performance in hikers and trapeze sailors were shown in Table 2. As can be seen hikers’ bilateral quadriceps isometric PT were significantly higher than trapezing sailors’(*p*<0.05). Male hikers’ bilateral quadriceps concentric and eccentric PT were significantly higher than male trapeze sailors’ (*p*<0.05), and there were no differences between female sailors. The parameters of CMJ performance observed in hikers were not significantly different from those of the trapeze sailors (*p*>0.05).

**Table 2:** Comparison in isokinetic strength and CMJ performance between hikers and trapeze sailors.

			Male		Female	
			Hikers (N = 11)	Trapeze sailors (N = 11)	Hikers (N = 15)	Trapeze sailors (N = 8)
Isokinetic Strength (N*m/kg)	L Q Con PT ^a^		3.12±0.15	2.97±0.09	2.78±0.08	2.81±0.09
	L Q Ecc PT ^a^		3.42±0.15	3.25±0.17	2.98±0.12	3.05±0.18
	L Q Iso PT ^ab^		3.31±0.14	3.15±0.13	2.94±0.10	2.85±0.07
	L H Con PT		1.62±0.12	1.53±0.10	1.29±0.14	1.34±0.18
	L H Ecc PT		1.86±0.15	1.73±0.09	1.50±0.15	1.61±0.26
	R Q Con PT ^a^		3.14±0.16	3.02±0.11	2.79±0.08	2.83±0.12
	R Q Ecc PT ^a^		3.48±0.15	3.32±0.20	3.00±0.14	3.09±0.16
	R Q Iso PT ^ab^		3.32±0.12	3.19±0.11	2.97±0.11	2.86±0.10
	R H Con PT		1.65±0.21	1.64±0.14	1.40±0.10	1.35±0.14
	R H Ecc PT		1.97±0.19	1.81±0.14	1.52±0.13	1.58±0.24
L Conventional H/Q ratio			0.55±0.10	0.52±0.03	0.52±0.03	0.46±0.05
R Conventional H/Q ratio			0.53±0.07	0.53±0.06	0.54±0.04	0.50±0.03
L Functional H/Q ratio			0.62±0.10	0.60±0.04	0.58±0.02	0.54±0.05
R Functional H/Q ratio			0.62±0.09	0.63±0.06	0.60±0.04	0.54±0.03
CMJ performance	Height (cm)		37.18±4.52	38.59±4.53	28.02±3.88	27.56±3.90
	Peak Force (N/kg)		237.5±19.9	239.9±18.2	215.6±14.0	209.9±14.1
	Peak Power (W/kg)		53.43±4.51	53.43±4.36	43.93±4.08	42.16±5.09

Note: L: left; R: right Q: quadriceps; H: hamstring; Con: concentric; Ecc: eccentric; Iso: isometric; PT: peak torque; a: significant difference between male hikers and male trapeze sailors (*p*<0.05); b: significant difference between female hikers and female trapeze sailors (*p*<0.05).

**Fig 1:**
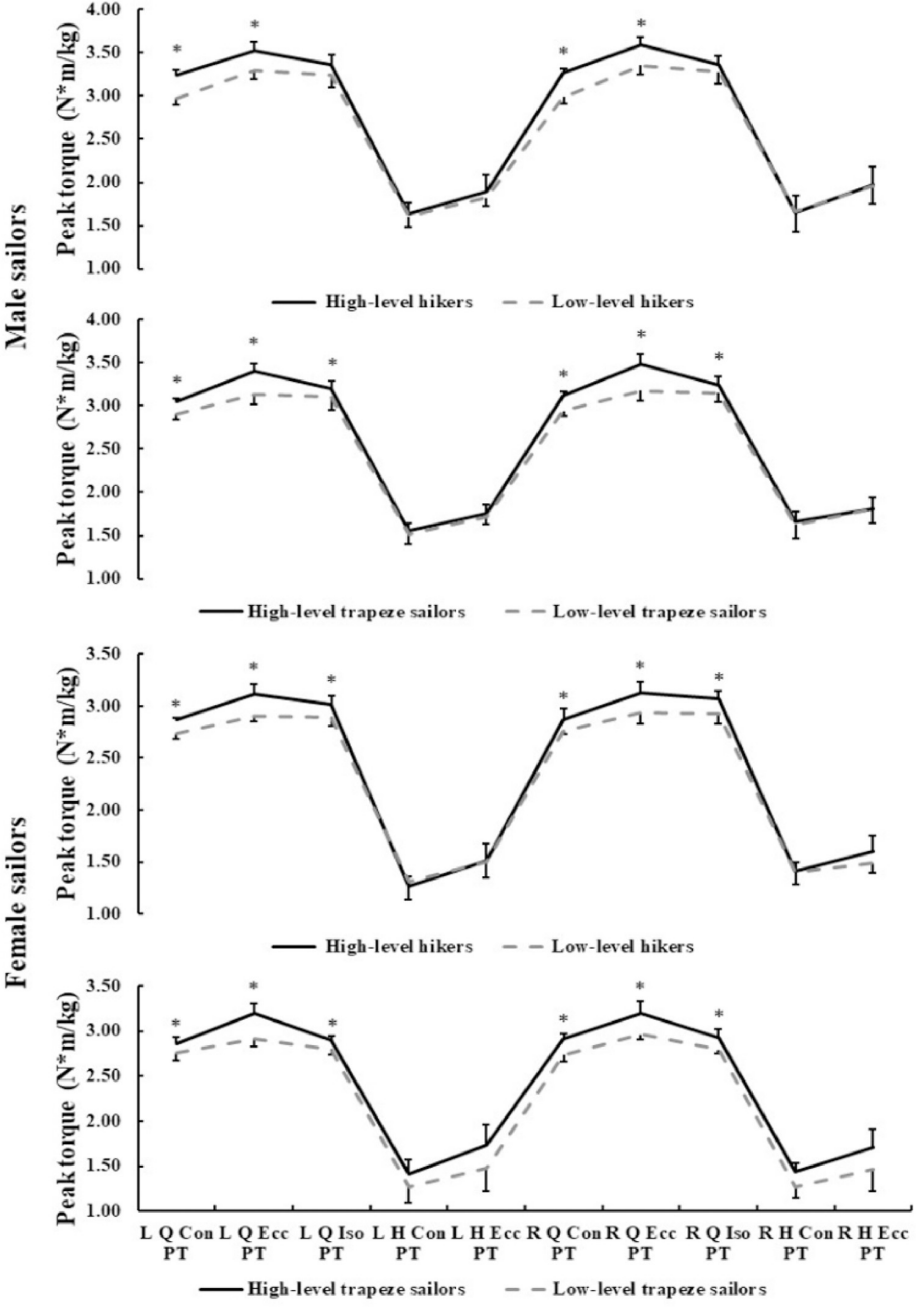
Comparison of peak torque in isokinetic muscle strength test between hikers and trapeze sailors at different levels. (* indicates significant difference between high-level and low-level sailors, *p*<0.05).

As we can see from Fig 1. For male hikers, the quadriceps concentric and eccentric PT in high-level sailors were significantly higher than those in low-level sailors, with the values of 3.24±0.07 vs 2.98±0.08 (N*m/kg), 3.52±0.11 vs 3.29±0.10 (N*m/kg), and 3.27±0.05 vs 2.98±0.07 (N*m/kg), 3.59±0.09 vs 3.35±0.11 (N*m/kg) in left and right legs, respectively (*p*<0.05). There was no difference in isometric PT of male hikers with different levels. For female hikers, however, not only the values of concentric and eccentric PT, the bilateral quadriceps isometric PT of high-level group were significantly higher than those in low-level group (left side: 3.01±0.08 vs 2.90±0.09 (N*m/kg), *p* = 0.013; right side: 3.08±0.07 vs 2.92±0.10 (N*m/kg), *p* = 0.013. For trapeze sailors, the concentric, eccentric and isometric PT of bilateral quadriceps of high-level group were higher than those in low-level group (*p*<0.05).

**Fig 2:**
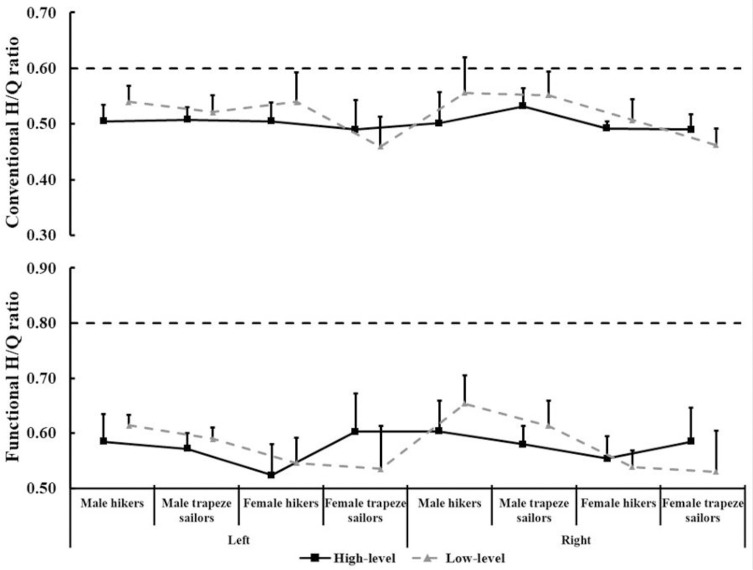
Conventional and functional isokinetic H/Q ratio.

As we can see from Fig 2. Based on PT conventional H/Q ratio ranged from 0.44 to 0.56 in all sailors. Based on PT functional H/Q ratio ranged from 0.52 to 0.65 in all sailors.

**Fig 3:**
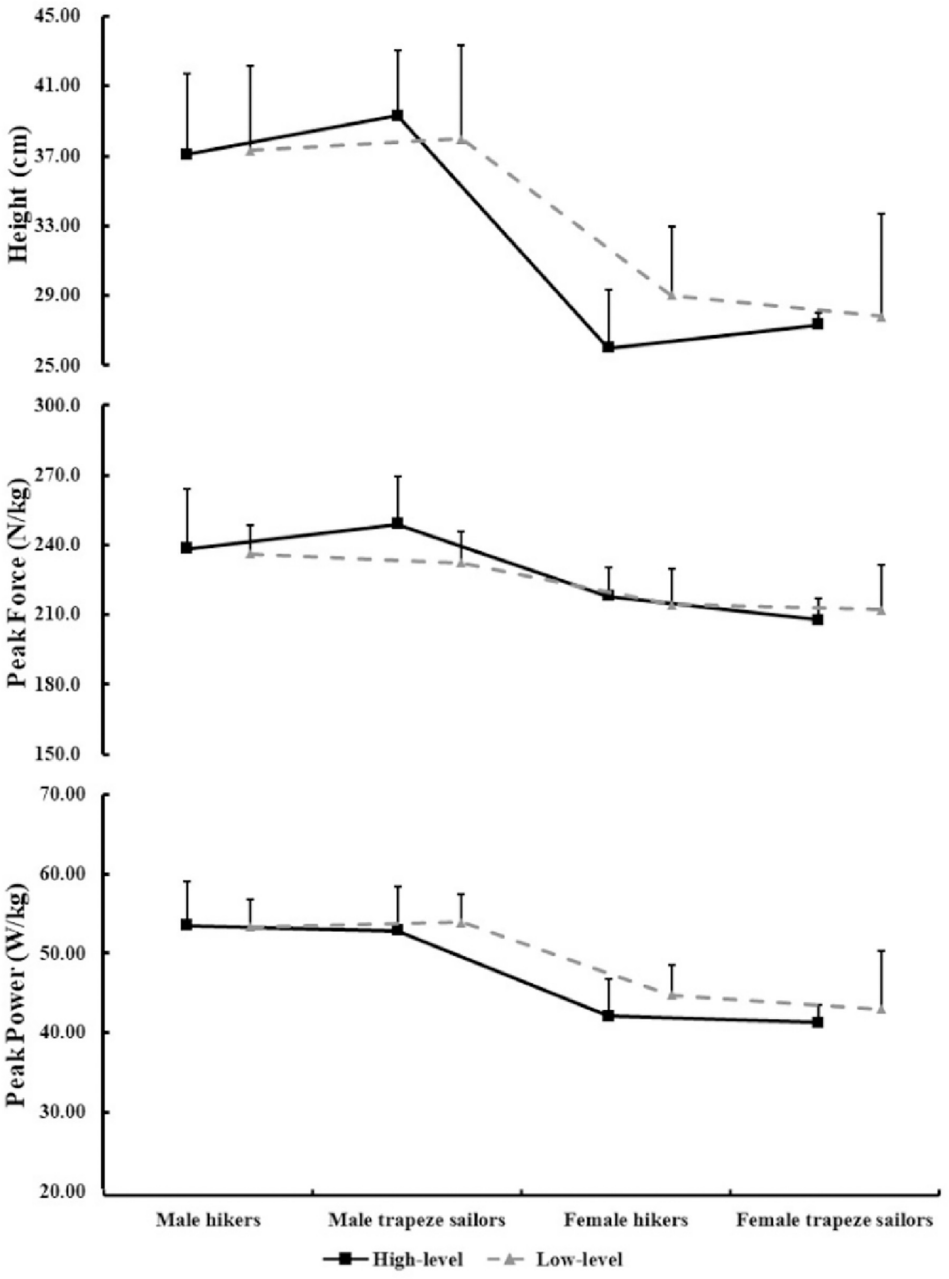
Comparison in CMJ performance between hikers and trapeze sailors at different levels.

It can be seen from Fig 3 that the parameters of CMJ performance (height, peak force and peak power) observed were not significantly different in high-level and low-level hikers and trapeze sailors (*p*>0.05).

The correlation coefficients among the measured variables were presented in Table 3. CMJ performance was moderately to strongly (r ranging 0.50–0.81) correlated with the isokinetic quadriceps PT (*p*<0.01).

**Table 3:** The correlation coefficient between quadriceps isokinetic strength and CMJ performance.

Parameters	Height	Peak Force	Peak Power
Concentric Peak Torque	0.67 ^+^	0.81 ^+^	0.80 ^+^
Eccentric Peak Torque	0.50 ^+^	0.60 ^+^	0.65 ^+^
Isometric Peak Torque	0.61 ^+^	0.80 ^+^	0.76 ^+^

Note: ^+^ *p* < 0.01.

## Discussion

The aims of this study were to analyze the characteristics and relationships of lower-limbs strength and power between hikers and trapeze sailors with different levels. Our hypothesis was that measures of isokinetic quadriceps and hamstrings PT, CMJ height, peak force and peak power would differentiate hikers and trapeze sailors with different levels.

The isokinetic knee flexor and extensor PT are applied to assess joint function and muscle strength at different angular speeds and are key effectors during high performance activities [12]. The slow speed test (namely 60°/s) is an important indicator for resistance and comprehensive strength [13]. In the process of isokinetic knee joint movement, the higher PT of the flexor and extensor, the greater muscle strength and the stronger work ability. In our study, hikers’ quadriceps isometric PT was significantly higher than trapeze sailors’, and male hikers had a higher concentric and eccentric quadriceps PT at 60°/s angular velocity, but no difference between hamstrings. Hiking activity involves holding the hips, trunk, head, and upper extremities in sustained and unsupported positions over the side of a boat, which requires the hikers to use internal strength of the body to resist the roll of the boat, the muscles most loaded are the quadriceps of the thigh [14, 15]. In the hiking process, the quadriceps has near-isometric exercise in different duration, so the isometric contraction force of the quadriceps is one of the key factors affecting hiking performance [2, 16]. The muscles with higher strength level may reduce the relative isometric contraction load of the hiking, allowing sailors to tolerate and maintain isometric activity in the most optimal conditions [17]. However, environmental factors such as wind and waves are constantly changing, sustained static hiking is extremely rare. In actual competition, sailors need to adjust their positions according to the environment and opponent changes, and knees, hips, and lumbar girdle continue to perform fast flexion and extension movements to optimal hiking moment [18]. Long-term lower limbs activities make hikers’ quadriceps have excellent concentric and eccentric strength.

The function of the trapezing is to enable the sailors to exert a righting force by getting their body further out of the deck. The physical stress placed on the sailor who is constantly swinging in and out on the wing of the boat via the trapezing. In moderate winds when twin wiring on the trapezing is intermittent, the crew normally counteracts the changes in wind strength by coming in and then going back out on the trapeze wire whereas the helm tries to remain as static as possible on the trapezing. As wind speed increases, trunk and knee extension also increase, causing the righting moment to change accordingly [19, 20]. In addition, trapezing techniques have become more dynamic than hiking, which involves a rapid flexion-extension movement of the spine, and include a large number of other physical movements such as trimming the spinnaker and pumping the main sail [21], while hiking technique pays more attention to the lower limbs muscle activities of sailors in different contracting forms. That is why we can observe that male hikers had greater lower limbs muscle strength than male trapeze sailors in our study.

The results of the study on female sailors showed that there was no difference in quadriceps concentric and eccentric PT between hikers and trapeze sailors. The reason may be that the height and weight of female sailors are lower than that of male, and the righting moment formed by the body is small. In addition, the strength of female sailors is relatively small, and they cannot control the boat with strength like male, so they are more inclined to resist changes in wind and waves through maneuvers, equipment and body position adjustments during sailing.

Long term specific training will lead to sport-specific muscle function differences. In the present study, the high-level sailors possessed greater concentric, eccentric and isometric quadriceps strength compared to low-level sailors. Aagaard et al. showed that eccentric knee extensor strength was higher in male high-level sailors compared to male control subjects, indicating that sailors had high level of maximum eccentric knee extensor strength [22]. Friedsenbichler and colleagues found knee extensor torque was higher in sailors compared to non-sailors under eccentric (+20%) and isometric (+14%) but not concentric conditions [23]. Highly-ranked compared to less well-ranked sailors demonstrated greater mean hiking moment and significantly prolonged hiking endurance time (298 ± 10 s vs 201 ± 7 s), which may because high-level sailors have superior cardiovascular and quadriceps oxygen utilization capacities [24]. Long-term training adaptation makes high-level sailors have better quadriceps strength and lower rate of neuromuscular fatigue, and optimal adjusting neural distribution of activities within and between muscles [25].

The H/Q ratio provides a quantifiable measurement of the torque from agonist and antagonist contraction of the knee joint, and is an important factor for evaluating the function of the knee. Traditional H/Q ratio, namely the concentric quadriceps PT is compared with the concentric hamstrings PT, ranges from 0.50 to 0.80 based on angular velocity, increased with angular velocity [26]. Generally, 0.6 is considered normal [27, 28]. In practice, the agonist quadriceps contract concentrically and the antagonist hamstrings contract eccentrically during leg extension, the ratio between them is dynamic control ratio or functional H/Q ratio. Ratio 1 is a suggested point of equality, which can better reflect the agonist-antagonist strength balance and dynamic knee stability [29].

The results of this study showed that the traditional and functional H/Q ratios of sailors at 60°/s angular velocity ranged from 0.44 to 0.56 and 0.52 to 0.65, respectively. Both ratios are lower than the recommended, indicating the strength of knee flexor and extensor in sailors is seriously unbalanced. The traditional H/Q ratio of sailors in our study was significantly lower than the dominant leg ratio of high-level Turkish Laser (0.66) and 470 class sailors (0.63) [30]. Studies have shown that H/Q ratio is relatively low in most sailors, and knee joints injury is also one of the most common injuries in sailing [31], which may be related to the sailors’ movements and the forms of muscle exertion. For hikers, hiking activity run through all part of the race, with the quadriceps being the main supporting muscle. Sailors constantly adjust their body positions according to the changes of wind force and wave conditions, the knee joint is in a continuous state of extension, and the quadriceps contraction may lead to substantial anterior translation and internal rotation of the tibia, thereby increasing the stress load of the anterior cruciate ligament [32]. Meanwhile, hikers’ anterior thigh muscles and trunk flexors undergo strong alternate isometric, concentric, eccentric contractions, which may cause rapid fatigue of these muscles and force sailors to isolate the vastus lateralis muscle while hiking, resulting in lateral tracking patella and resultant patella femoral knees pain [33]. Trapeze sailors also increase the angle of legs extension to gain greater righting torque, which will cause pressure on thigh extensor muscles [19]. In addition, sailors turn from one side of the boat to the other in tacking/gybing, the quadriceps produces a high PT. At this point, the co-activation of the hamstring muscle not only helps to recruit more quadriceps to generate additional extension torque [34], but also can effectively brake, increase the stability of the knee joint and reduce the incidence of injuries [28]. Therefore, the antagonist hamstrings strength should be properly strengthened in future training to improve the stability of knee joint and prevent injury.

The CMJ test is common in physical education and sports training, as a means to assess lower limbs power and neuromuscular function. There was positive correlation between CMJ height with the knee extensor PT, individuals with higher lower limbs strength level have higher peak power when performing various jumping movements were reported by Uslu et al. [35, 36]. However, we failed to arrive at similar results, the parameters of CMJ have no difference in hikers and trapeze sailors, as well as high-level and low-level groups. Both kinetic and kinematic factors during the jump are important determinants of CMJ performance, on the one hand, the adaptation of neuromuscular tissue to strength and power training is improved, and on the other hand, technique influencing the extent to which maximal muscle capabilities can be utilized during the jump [8]. The CMJ transmits the reaction force of the ground to the longitudinal direction by extending the hips, knees and ankles. The maneuvers of sailing, whether hiking or trapezing, basically do not involve the extension of the ankle joint. Sailors take the foot as a support point and exercise by flexing and extending the knees and hips, which are quite different from the CMJ motion model. Therefore, although sailors have well enough lower limbs strength, it does not show the difference in CMJ performance.

A number of studies have examined the relationship between isokinetic knee extensor PT and jumping performance, and the results are quite different. In this study, there was a moderate-strong correlation between the eccentric and isometric PT of quadriceps and CMJ performance, and a stronger correlation between the quadriceps concentric PT and CMJ parameters. The CMJ activity utilize the stretch shortening cycle (SSC) to enhance performance. During the SSC the athlete commences a counter movement by first relaxing the agonist muscles, the quadriceps muscle is stretched eccentrically, which results in enhanced force production during the subsequent concentric phase [37]. Some studies have examined that the jump performance-isokinetic knee extensor muscles strength relationship depended on the angular velocity, with no or low correlation at slow angular velocities (lower than 180°/s) and a moderate-high correlation at fast angular velocities [36]. Malliou and Ispirlidis stated that there was a significant correlation between the knee extensor PT at 60°/s and CMJ height for professional soccer players after the completion of the competition and preparation period (r ranging 0.430–0.587) [38]. Pääsuke et al. found Nordic combined athletes and untrained male university students CMJ height and isokinetic knee extension PT at angular velocities of 60°/s (r = 0.88 and r = 0.7) and 180°/s (r = 0.69, r = 0.59) had positive correlation [39]. Tsiokanos reported correlation coefficients of 0.57, 0.64 and 0.36 between CMJ height and isokinetic knee extension torque at 60°/s, 120°/s and 180°/s, respectively, in an untrained group [40]. These differences may be related to diagnostic methods, training level, CMJ technique, sport-specific characteristics and other factors. This study did not measure other angular velocities, so the correlation between knee extensor muscle PT and CMJ at different angular velocities of sailors could not be identified.

## Study limitations

The study limitations include the isokinetic testing was conducted only at 60°/s angular velocity. If we conducted more studies of the isokinetic testing at different velocities, we might have more relevant results. Furthermore, the number of hikers and trapeze sailors at different levels is small, so future studies should increase the participant sample to better discover the differences of sailors.

## Practical applications

Our findings provide an important reference for the development of lower limbs strength training programs for sailors. High-level sailors exhibit highly developed characteristics across a range of lower limbs muscle strength measures, implying that training programs should pay more attention to the strength development of the quadriceps in sailors, especially with hikers. The strength of lower limbs hamstrings and quadriceps of sailors is unbalanced, and H:Q ratio is significantly lower than the recommended value, there is a risk of injury. In future training, the hamstrings muscle strength should be properly strengthened to improve the stability of knee joint and prevent injury.

## Conclusions

According to our study, long-term training can induce specificity of sport in lower limbs strength of sailors, and there are differences between hiker and trapeze sailors. Hikers have better quadriceps isometric strength compared to trapeze sailors. The quadriceps concentric and eccentric peak torque can be used as parameters to distinguish between high-level and low-level sailors, but CMJ cannot.

## Supporting information

S1 Dataset (XLSX)

## Abbreviations

CMJ: Counter movement jump
PT: Peak torque
H/Q: Hamstring / quadriceps
Con: Concentric
Ecc: Eccentric
Iso: Isometric
Q: Quadriceps
H: Hamstring
